# Evaluating lignin degradation under limited oxygen conditions by bacterial isolates from forest soil

**DOI:** 10.1038/s41598-024-64237-8

**Published:** 2024-06-10

**Authors:** Thitinun Sumranwanich, Esther Amosu, Surang Chankhamhaengdecha, Tanaporn Phetruen, Wethaka Loktumraks, Puey Ounjai, Phurt Harnvoravongchai

**Affiliations:** 1 Department of Biology, Faculty of Science, Mahidol University, Thung Phaya Thai, Ratchathewi, Bangkok, 10400 Thailand; 2 Department of Biochemistry, Faculty of Science, Mahidol University, Thung Phaya Thai, Ratchathewi, Bangkok, 10400 Thailand

**Keywords:** Forest soil, Lignocellulosic biomass, Oxygen limited condition, Lignin degrading bacteria, Biotechnology, Microbiology, Environmental sciences

## Abstract

Lignin, a heterogeneous aromatic polymer present in plant biomass, is intertwined with cellulose and hemicellulose fibrils, posing challenges to its effective utilization due to its phenolic nature and recalcitrance to degradation. In this study, three lignin utilizing bacteria, *Klebsiella* sp. LEA1, *Pseudomonas* sp. LEA2, and *Burkholderia* sp. LEA3, were isolated from deciduous forest soil samples in Nan province, Thailand. These isolates were capable of growing on alkali lignin and various lignin-associated monomers at 40 °C under microaerobic conditions. The presence of Cu^2+^ significantly enhanced guaiacol oxidation in *Klebsiella* sp. LEA1 and *Pseudomonas* sp. LEA2. Lignin-related monomers and intermediates such as 2,6-dimethoxyphenol, 4-vinyl guaiacol, 4-hydroxybenzoic acid, benzoic acid, catechol, and succinic acid were detected mostly during the late stage of incubation of *Klebsiella* sp. LEA1 and *Pseudomonas* sp. LEA2 in lignin minimal salt media via GC–MS analysis. The intermediates identified from *Klebsiella* sp. LEA1 degradation suggested that conversion and utilization occurred through the β-ketoadipate (*ortho*-cleavage) pathway under limited oxygen conditions. The ability of these bacteria to thrive on alkaline lignin and produce various lignin-related intermediates under limited oxygen conditions suggests their potential utility in oxygen-limited processes and the production of renewable chemicals from plant biomass.

## Introduction

Lignocellulosic biomass is an abundant, low-cost, and renewable source for the production of biofuels and other substitutes for petroleum-derived chemicals^1^. The major components of lignocellulose are cellulose (40–60%), hemicellulose (20–40%), and lignin (10–25%)^2^. Lignin is a heterogeneous aromatic polymer, predominantly interwoven with cellulose and hemicellulose in the plant cell walls, and has been identified as a key factor impeding the effective utilization of lignocellulosic biomass. Its complex aromatic structure, along with stable carbon-to-carbon and ether linkages, confers resistance to chemical and biological degradation, thus posing challenges to decay^3–5^. Hence, the removal of lignin is crucial for improving the breakdown of plant biomass to allow access to cellulose and hemicellulose in the plant cell wall. Conventional methods for lignin removal from lignocellulose include steam explosion, organosolv treatment, and alkali or acid hydrolysis, among others^6–8^. However, these methods are associated with high costs, energy consumption, and the production of toxic phenolic compounds as undesirable byproducts^5^. The utilization of microorganisms in biological delignification for biomass pretreatment emerges as an ecologically friendly alternative, requiring minimal use of chemicals and energy^8^.

Globally, approximately 100 million tons of lignin are produced annually from lignocellulosic biomass, primarily through various pretreatment methods. The pulp and paper industry alone generates 50–70 million tons of lignin as a byproduct annually^9^. With the growing interest in the utilization of lignocellulosic biomass for biofuel production, there is a potential surge in annual lignin production^9^. Therefore, it becomes imperative to develop strategies for lignin valorization, rather than resorting to its mere combustion for fuel, as commonly practiced in pulp industries. Notably, the heterogeneous and aromatic compositions of lignin make it a potentially abundant source for various renewable or low molecular weight chemicals used in plastics production, as well as in pharmaceuticals, food, flavour, and aroma industries^10–12^. Extensive studies have demonstrated the ability of microorganisms, particularly fungi, to degrade lignin in plant structure^13–16^. Moreover, microbial ligninolytic enzymes have been reported to effectively decolourize aromatic organic and synthetic dyes found in wastewater effluents of textile industries^17–20^. Therefore, employing microorganisms to develop systems capable of efficiently breaking down lignin structures into smaller and less toxic components would be highly advantageous, not only for chemical extraction but also for remediating toxic wastes released into the environment.

Apart from fungi, lignin-degrading genes and enzymes are also present in bacteria isolated from various sources such as soil samples, rotten wood, termite guts, and coal^5,12,20–22^. Bacteria offer several advantages over fungi in the process of lignin degradation, owing to faster growth rates, broader tolerance of diverse growth conditions and habitats, facilitation of genetic manipulation, and efficient protein expression. Furthermore, large scale bacterial cultivation is potentially more efficient in terms of management^17^. Examples of reported bacteria and their enzymes associated with native lignin or lignin related compounds degradation include *Sphingobium* sp. SYK-6, *Pseudomonas putida* mt-2, *Rhodococcus jostii* RHA1, *Acinetobacter* sp., *Streptomyces coelicolor*, *Microbacterium* sp., *Enterobacter ligninolyticus* SCF1*, Burkholderia* sp. H1, *Bacillus pumilus*, and *Bacillus atrophaeus*^6,23–27^. Meanwhile, extensive studies on bacterial lignin degradation have been conducted under aerobic conditions, and fewer studies have explored the ability of anaerobic or facultative anaerobic bacteria to degrade lignin^28–30^. Given that limited oxygen is a common challenge in several treatment systems, bacteria capable of utilizing lignin under oxygen limitation would offer significant advantages in numerous applications.

Although different lignin-degrading bacteria have been isolated and identified, there is still a gap in the knowledge of utilization involved in lignin metabolism. The mechanisms of ligninolytic enzymes are also yet to be characterized. Application of microbial ligninolytic enzymes is still limited and not sufficient for large-scale or industries. Therefore, exploring lignin-degrading bacteria that may harbour novel or more effective enzymes for metabolizing its derivatives, particularly under oxygen limited conditions, may provide a better opportunity for improving industrial processes.

Here, the soil bacteria that display the ability to grow on alkali lignin under microaerobic conditions were isolated from tropical deciduous forest due to its richness in microbial diversity as well as the potential presence of active lignocellulolytic microorganisms^31,32^. Alkali lignin was used as the model to study lignin utilization in this study. Three newly identified alkali lignin utilizing bacteria were successfully isolated from soil samples collected from the deciduous forest in Nan, northern Thailand. Observations of lignin transformation in microaerobic conditions were present, and proposed pathways for the breakdown of lignin were delineated using the derived products.

## Results

### Bacteria isolation and identification

**Figure 1:**
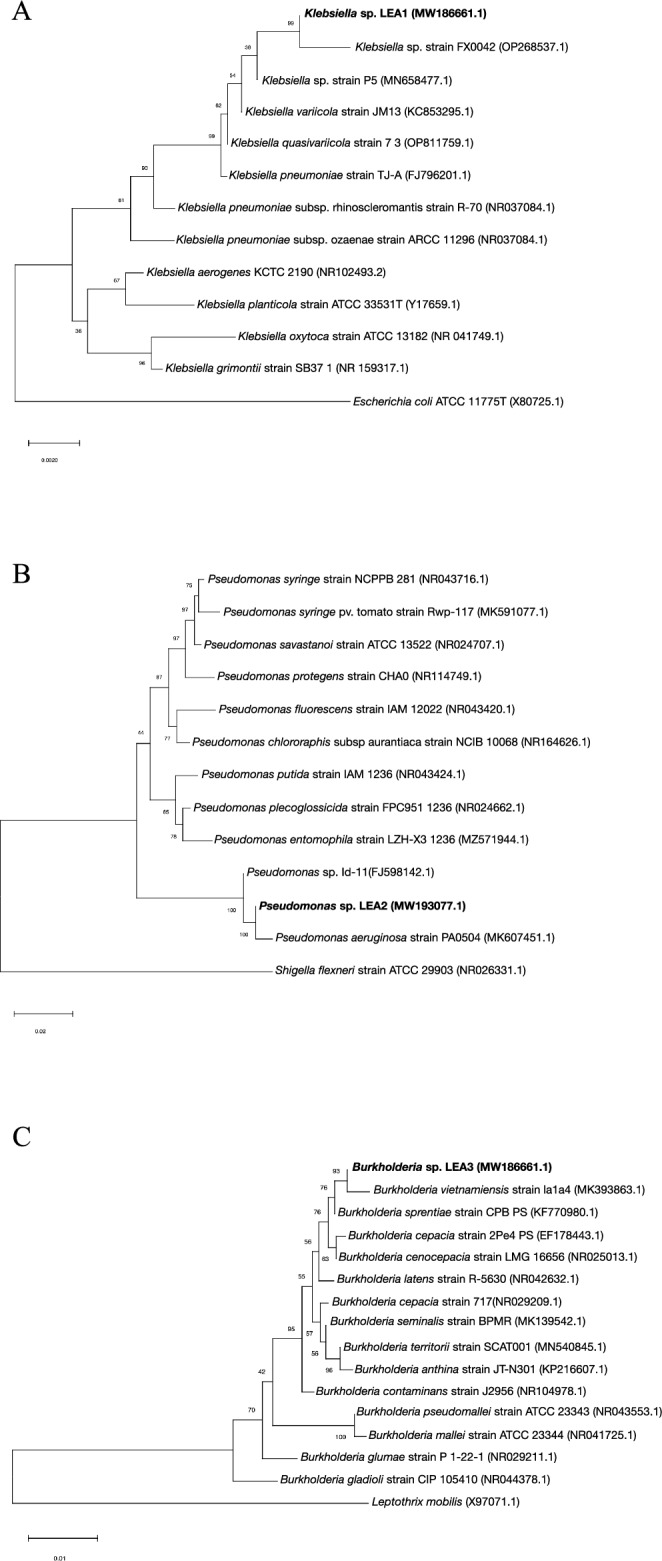
Phylogenetic tree of 16S rDNA The tree showing the taxonomic relationship of the three isolates, (**A**) *Klebsiella* sp. LEA1, (**B**) *Pseudomonas* sp. LEA2, and (**C**) *Burkholderia* sp. LEA3 with the bacteria in the same genus. The isolates in this study are marked in bold.

A total of 16 bacterial colonies with different colony sizes and morphologies were selected from the lignin enrichment process. The selected bacterial isolates were those that exhibited utilization or tolerance to the presence of phenolic compounds prevalent in lignin under limited oxygen conditions. For initial screening, colonies that grew on L-MSM (Lignin minimal salt media) with alkali lignin as the sole carbon source were selected for further screening. Afterward, the selected colonies were streaked onto carboxymethyl cellulose (CMC) agar plates to further select colonies with the ability to utilize cellulose as an energy source in addition to lignin. Nine bacterial isolates were identified by 16S rDNA sequencing to be closely related to *Klebsiella* sp., four were closely related to *Burkholderia* sp.*,* and three to *Pseudomonas* sp., (Supplementary Table S1). The reported growth of the bacteria is consistent with the previous evidence in that all three genera possess lignin degrading ability^33,34^. Selected representative isolates, *Klebsiella* sp. LEA1, *Pseudomonas* sp. LEA2 and *Burkholderia* sp. LEA3 (16S rDNA sequences are available in the NCBI database with accession numbers MW186661, MW193077, and MW193078, respectively) were chosen for further characterization. The phylogenetic trees of each isolate were constructed along with the bacteria in the same genus retrieved from the NCBI database. According to the phylogenetic trees, *Klebsiella* sp. LEA1 is the most closely related to *Klebsiella* sp. FX0042 isolated from the soil in China. The bacterium is also grouped into the same clade with *Klebsiella* sp. P5 and *K. variicola* JM13 (Fig. 1A)*.* For *Pseudomonas* sp. LEA2 and *Burkholderia* sp. LEA3, the taxonomic analysis revealed the closest relationship of the bacteria to *P. aeruginosa* PA0504 and *B. vietnamiensis* la1a4, respectively (Fig. 1B,C).

### Growth on lignin monomers and effect of copper (Cu^2+^) on guaiacol oxidation

**Figure 2:**
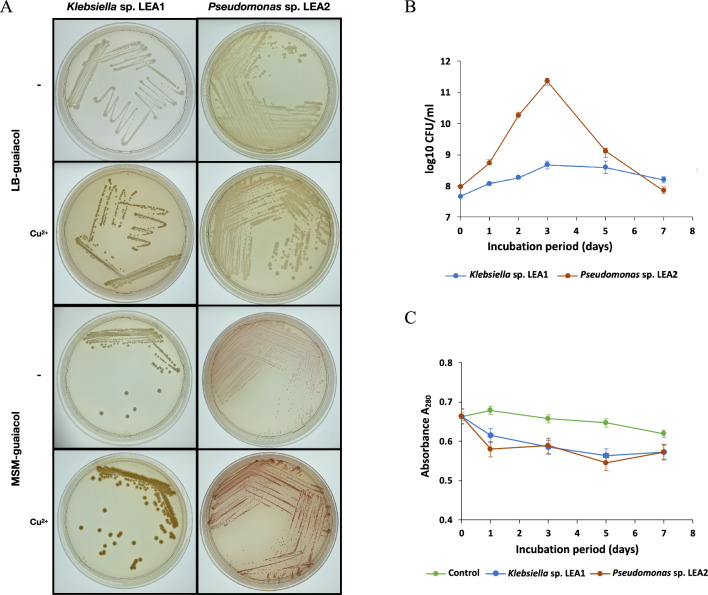
Bacterial growth and oxidation of guaiacol (**A**) Guaiacol oxidation with the presence of Cu^2+^. Effects of Cu^2+^ on guaiacol oxidation on minimal salt media (MSM) and LB media supplemented with 0.1% (v/v) guaiacol. Red/brown pigmentation around bacteria colonies indicates guaiacol oxidation in the media. Guaiacol oxidation experiments were carried out in triplicates (Supplementary Fig. 1). (**B**) Growth of *Klebsiella* sp. LEA1 and *Pseudomonas* sp. LEA2 in L-MSM. The results of CFU/mL were calculated from triplicate samples. (**C**) Decrease in total phenolic content (A_280_) in the sample treated with *Klebsiella* sp. LEA1 and *Pseudomonas* sp. LEA2. Cultures were incubated at 40 °C for 7 days under microaerobic conditions. The absorbance of 280 nm was calculated from three independent experiments.

In preliminary experiments, *Klebsiella* sp. LEA1 and *Pseudomonas* sp. LEA2 were able to grow on minimal salts media agar plates containing three types of model lignin monomers (guaiacol, veratryl alcohol, and 2,6-Dimethylphenol (2,6 DMP))^35–37^ as the sole carbon source, while the growth of *Burkholderia* sp. LEA3 was only observed on veratryl alcohol and 2,6 DMP. No growth observation was found for *Burkholderia* sp. LEA3 on MSM supplemented with guaiacol (Supplementary Table S2). For nutritionally rich medium, the isolates could grow on LB media supplemented with guaiacol except for *Burkholderia* sp. LEA3, which exhibited no growth. Copper (Cu^2+^) has been reported to be present at the active site of laccase enzyme^38^, therefore, improvement of lignin-degrading ability with the addition of Cu^2+^ was determined. *Klebsiella* sp. LEA1 and *Pseudomonas* sp. LEA2 showed enhanced guaiacol oxidation with the presence of Cu^2+^ in the guaiacol medium (Fig. 2A). Oxidation was greatly increased when the bacteria were grown on LB based medium. Guaiacol is a major aromatic monomer found in depolymerized lignin; therefore, *Klebsiella* sp. LEA1 and *Pseudomonas* sp. LEA2 were selected for further analyses based on the oxidation activity.

### Growth characteristic and alkaline lignin utilization of the isolates

Growth of *Klebsiella* sp. LEA1 and *Pseudomonas* sp. LEA2 in alkali lignin containing L-MSM was observed over a period of 7 days (Fig. 2B). Although *Pseudomonas* sp. LEA2 and *Klebsiella* sp. LEA1 were initially inoculated at an equivalent OD_600_ of 0.1 with slightly different inoculum (9.20 ± 0.85 × 10^7^ and 4.52 ± 0.35 × 10^7^ CFU/mL, respectively), *Pseudomonas* sp. LEA2 exhibited an accelerated growth rate in the first 3 days compared to *Klebsiella* sp. LEA1. Both isolates exhibited steady growth and reached their maximum cell density at day 3 of incubation, with the bacterial density of 2.29 ± 0.52 × 10^11^ and 4.75 ± 1.39 × 10^8^ CFU/mL for *Pseudomonas* sp. LEA2 and *Klebsiella* sp. LEA1, respectively. The decline in growth was observed after the third day, suggesting lignin tolerance and the utilization of alkaline lignin or degraded products as a carbon source. Concurrently, the total phenolic compound was monitored through the reduction in absorbance at 280 nm (Fig. 2C). Both isolates exhibited a pronounced decrease in detected absorbance, indicating a reduction in total phenolic content. Given the potential influence of various molecules and lignin internalization by the bacteria on A280, additional experiments were subsequently conducted to conclusively support lignin-degrading ability.

**Figure 3:**
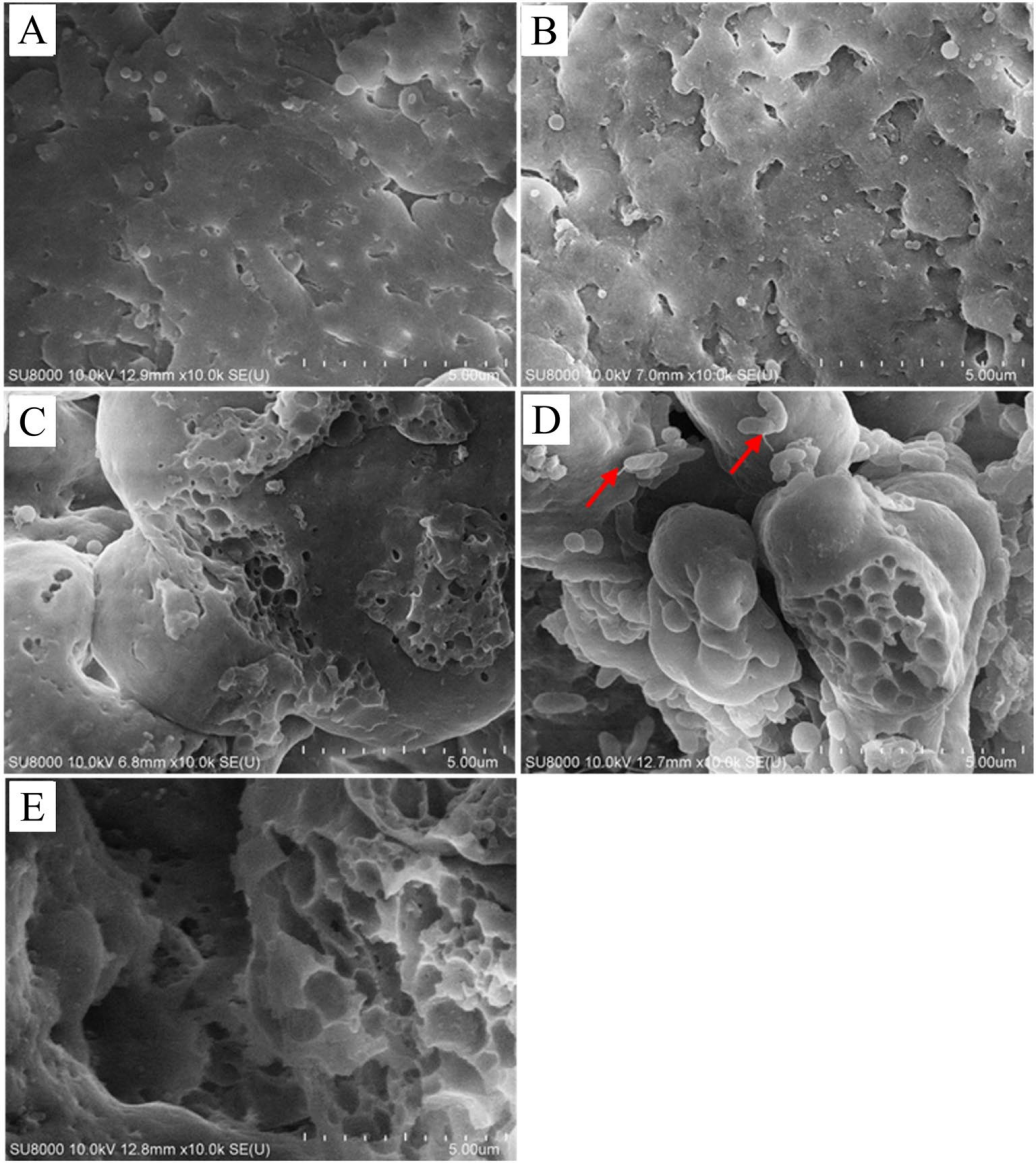
SEM images of lignin samples. (**A**) Control sample day 0 (**B**) Control sample day 7 (**C**) Lignin sample treated with *Klebsiella* sp. LEA1 day 7 (microaerobic condition) (**D**) Lignin sample treated with *Pseudomonas* sp. LEA2 day 7 (microaerobic condition) (**E**) Lignin sample treated with *Klebsiella* sp. LEA1 day 7 (aerobic condition). Arrows indicate bacterial cells on lignin sample.

**Figure 4:**
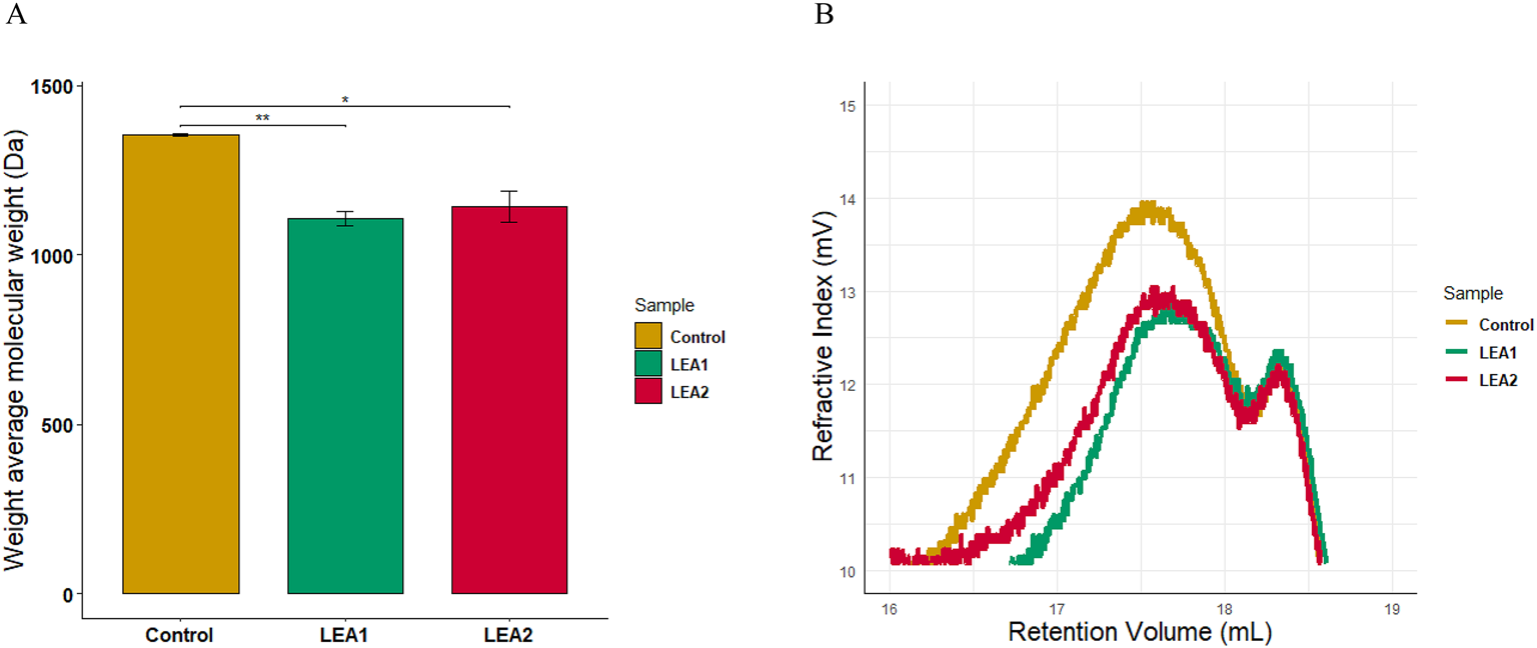
Capacity of the isolates to utilize lignin. (**A**) Average molecular weight of alkaline lignin. (**B**) GPC chromatogram of alkaline lignin. Three independent experiments were conducted (Supplementary Fig. 2), which one of the replicates is shown in (**B**). P < 0.05*, P < 0.01**.

The surface morphology of the lignin samples was also investigated using a scanning electron microscope (SEM) to assess the potential lignin-degrading ability of the bacteria. SEM images revealed that while control samples appeared relatively intact; samples treated with *Klebsiella* sp. LEA1 and *Pseudomonas* sp. LEA2 exhibited rough and porous surfaces after 7 days of incubation under aerobic and oxygen limited conditions (Fig. 3), suggesting potential lignin degradation. Gel permeation chromatography (GPC) was further employed to demonstrate the potential lignin-depolymerizing ability of the bacteria. Samples of alkali lignin after a 7-day incubation with *Klebsiella* sp. LEA1 and *Pseudomonas* sp. LEA2 in L-MSM under microaerobic conditions were extracted, and the results are summarized in Table 1. Changes in molecular weight distribution were supported by a reduction in number average molecular weight (Mn) from 791.7 ± 7.5 in the control sample to 714.0 ± 9.2 in *Klebsiella* sp. LEA1 and 734.3 ± 24.8 in *Pseudomonas* sp. LEA2. The control alkaline lignin displayed a weight average molecular weight (Mw) of 1353.7 ± 3.1 Da over a 7-day incubation period. In contrast, the samples treated with *Klebsiella* sp. LEA1 and *Pseudomonas* sp. LEA2 exhibited a significant decrease in the Mw of alkaline lignin, reaching 1107.3 ± 20.2 Da and 1142.7 ± 44.6 Da, respectively (Fig. 4A). The shift of the peak towards the higher retention time side also demonstrated potential depolymerization of alkaline lignin by the bacterial isolates (Fig. 4B). A decrease in the polydispersity index indicates a limited molecular weight distribution of the alkaline lignin, potentially resulting from the depolymerization of large lignin molecules. Considering the observations of the reduction in Mw, Mn, along with peak shift, could suggest capacity of lignin degradation or utilization by the bacterial isolates.

**Table 1:** Molecular weights distribution of alkaline lignin treated with the bacterial isolates.

Sample	Mn (Dalton)	Mw (Dalton)	Mz (Dalton)	Polydispersity (Mw/Mn)
Control	791.7 ± 7.5	1353.7 ± 3.1	2252.3 ± 30.4	1.711 ± 0.014
*Klebsiella* sp. LEA1	714.0 ± 9.2	1107.3 ± 20.2	1703.0 ± 32.5	1.564 ± 0.035
*Pseudomonas* sp. LEA2	734.3 ± 24.8	1142.7 ± 44.6	1744.0 ± 82.4	1.556 ± 0.011

### Analysis of alkaline lignin degradation products

GC–MS analysis was performed to identify products resulting from lignin utilization by both *Klebsiella* sp. LEA1 and *Pseudomonas* sp. LEA2 at days 3 and 7 of incubation (Table 2, Supplementary Fig. 3). Similar intermediates and products such as 4-vinyl guaiacol, isovanillyl alcohol, and 3-vanillyl propanol were detected in both isolates. Additionally, compounds such as phenol, 3,5-dimethyl cyclopentenolone, vanilonitrile, 2,6-dimethoxyphenol, vanillyl alcohol, eicosanoic acid were exclusively detected in *Pseudomonas* sp. LEA2, while several unique compounds such as pentanoic acid, cinnamic acid, and 4-hydroxybenzoic acid were only observed in *Klebsiella* sp. LEA1. Differences in lignin-derived intermediates and products between the two isolates suggest the employment of different pathways in lignin degradation.

**Table 2:** GC–MS product analysis of control and degraded samples under microaerobic conditions.

No	R.T	Compound	Control		*Klebsiella* sp. LEA1		*Pseudomonas* sp. LEA2	
			Day 0 and 3	Day 7	Day 3	Day 7	Day 3	Day 7
1	7.111	Lactic acid	−	+	−	−	−	+
2	8.014	Isovaleric acid	−	+	−	−	−	−
3	8.015	Pentanoic acid	−	−	−	+	−	−
4	9.294	Butanoic acid	−	+	−	+	−	−
5	10.598	Phenol	−	−	−	−	−	+
6	10.715	3,5-dimethyl cyclopentenolone	−	−	−	−	−	+
7	11.327	Guaiacol	+	+	+	+	+	+
8	13.211	Catechol	−	−	−	+	−	−
10	13.910	Benzoic acid	−	+	−	+	−	+
11	14.676	Benzeneacetic acid	−	+	−	+	−	+
12	14.924	Succinic acid	−	−	−	+	−	−
13	14.952	4-Vinyl guaiacol	−	−	+	−	−	+
14	16.130	2,6-Dimethoxyphenol	−	−	−	−	−	+
15	16.315	Benzenepropanoic acid	−	+	−	+	−	−
16	16.820	Vanilonitrile	−	−	−	−	+	+
17	16.895	Vanillyl alcohol	−	−	−	−	−	+
18	16.919	Isovanillyl alcohol	−	−	+	−	+	−
19	17.661	Vanillin	+	+	+	−	−	+
20	17.934	Cinnamic acid	−	−	−	+	−	−
21	18.274	Vanillic acid	+	+	+	+	+	+
22	18.924	4-hydroxybenzoic acid	−	−	−	+	−	−
23	19.209	Dihydro- coniferyl alcohol	+	+	+	+	+	+
24	19.889	Homovanillyl alcohol	+	−	+	−	+	+
25	20.332	Vanillyl ethyl ether	−	+	−	+	−	+
26	21.064	3-Vanillylpropanol	−	−	−	+	−	+
27	26.061	Eicosanoic acid	−	−	−	−	−	+
28	26.321	Hexanedioic acid, bis(2-ethylhexyl) ester	−	−	−	−	−	+

*Klebsiella* sp. LEA1 and *Pseudomonas* sp. LEA2 were grown in L-MSM under microaerobic conditions at 40 °C for 7 days. “ + ”, compound was detected; “−”, compound was not detected in culture; R.T: Retention time.

**Figure 5:**
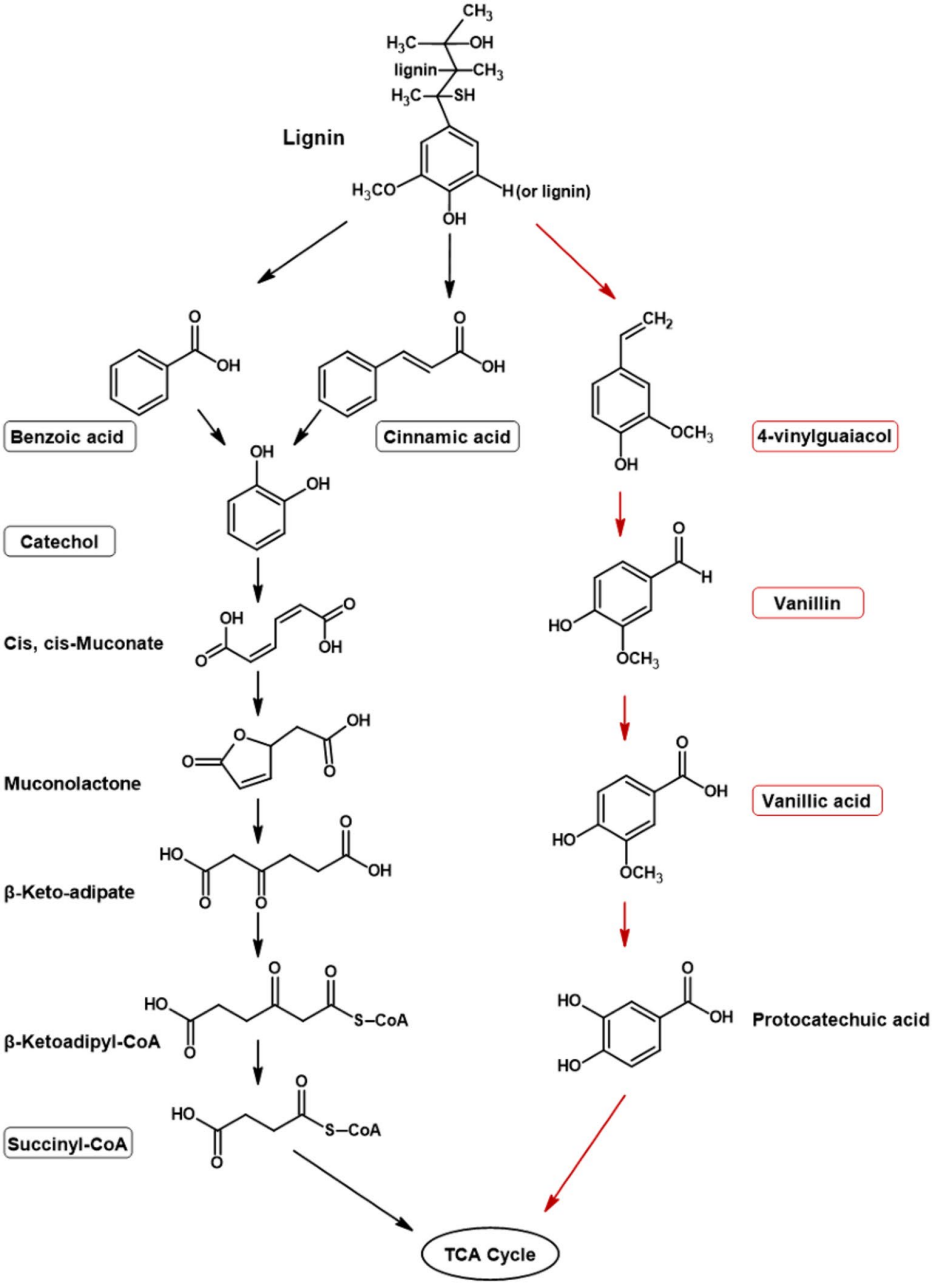
Proposed lignin degradation pathways in *Klebsiella* sp. LEA1. The pathway with black arrows represents the proposed *ortho*-cleavage of catechol through the β-ketoadipate pathway in *Klebsiella* sp. LEA1 under microaerobic conditions. The pathway with red arrows represents the proposed degradation pathway in *Klebsiella* sp. LEA1 under aerobic conditions. The pathways were developed based on metabolites detected in GC–MS in this study. Lignin related compounds and metabolites detected in the GC–MS profile are marked in boxes.

On day 7 of incubation under microaerobic conditions, certain organic acids such as butanoic acid, benzoic acid, and benzenepropanoic acid, which were absent on day 0 or 3 of incubation, were detected in the control samples. Although this indicates some degree of abiotic degradation in the control samples, a clear distinction was observed among the detected compounds in samples of *Klebsiella* sp. LEA1 and *Pseudomonas* sp. LEA2 compared to the control at day 7, supporting the presence of biotic degradation in the samples (Table 2). Despite an array of intermediates and products detected in both samples on day 7, none of the isovaleric acid, butanoic acid, benzenepropanoic acid or vanillin (present in control samples) were found in the samples inoculated with either of the two bacterial isolates, suggesting the utilization of the lignin-related compounds by the bacteria. Furthermore, the presence of benzoic acid, cinnamic acid, and catechol hinted at the use of the catechol branch of the β-ketoadipate pathway (*ortho*-cleavage) in *Klebsiella* sp. LEA1 under oxygen limited conditions (Fig. 5). Different lignin intermediates were identified in samples inoculated with *Pseudomonas* sp. LEA2 by GC–MS analysis as mentioned earlier (Table 2). However, no obvious indicator compounds or intermediates provides guidance for the pathway likely utilized by the isolate.

The dependence of lignin-degrading enzymes on oxygen has been previously reported^39^. Therefore, degradation products of lignin in *Klebsiella* sp. LEA1 were also investigated under aerobic conditions. In contrast to the microaerobic condition, most phenolic aromatics or lignin monomers such as benzeneacetic acid, vanillin, homovanillyl alcohol, and 4-vinyl guaiacol present in the control samples were not detected in the inoculated samples by day 3 of incubation (Table S3). By day 7, guaiacol and coniferyl alcohol were absent in the sample. Interestingly, while some lignin-related compounds were no longer detected in the sample at day 7, vanillic acid was retained throughout the incubation period (Table S3). This suggests that guaiacol degradation was greatly improved under aerobic conditions.

## Discussion

Lignin-degrading bacteria such as *Enterobacter ligninolyticus, Burkholderia* sp. H1, *Bacillus pumilus*, and *Bacillus atrophaeus* have previously been reported from soil samples^6,26,27,31^. Most lignin-degrading bacteria belong to three major classes: Actinomycetes, α-Proteobacteria, and γ-Proteobacteria^33^. Tropical forest soils have a rich microbial diversity and active lignocellulolytic microorganisms responsible for a high rate of plant litter decomposition compared to other biomes^31,32,40^. The genera of isolates obtained in this study (*Klebsiella*, *Burkholderia*, and *Pseudomonas*) showed consistency with the previous report of lignin-converting bacteria^33^. Alkali lignin and other model lignin compounds have generally been used to specifically isolate ligninolytic bacteria. Studies have also shown that microorganisms isolated on alkali lignin are able to degrade lignin present in plant biomass even though technical lignin varies in structure compared to native lignin^5,38^. The presence of these isolates in samples from different parts of Nan forest indicates their active or major involvement in lignin degradation in the environment. The ability of the isolates to grow on lignin-containing medium indicated depolymerization and their utilization of degraded compounds derived from alkaline lignin. Due to the phenolic nature of lignin, which exerts toxicity on most microorganisms by damaging cell membranes, enzymes, and nucleic acids, the ability to grow on lignin-containing media could also indicate the bacteria’s capability to tolerate lignin^41^. Isolates used in this study have the potential to utilize cellulose as an additional energy source based on their ability to grow on CMC plates. This is a valuable potential for lignocellulose-degrading bacteria as lignin and cellulose are major constituents of lignocellulosic biomass.

Based on the phylogenetic tree of 16S rDNA, *Klebsiella* sp. LEA1 shares a high sequence similarity with the soil bacterium *Klebsiella* sp. strain FX0042 and *Klebsiella* sp. strain P5 isolated from China. The isolate was also found to be closely related to the species-annotated *K. variicola* strain JM13 from plant rhizosphere. For *Pseudomonas* sp. LEA2*,* our bacterium shares the same node with soil isolated *P. aeruginosa* strain PA0504 from China. Likewise, *Burkholderia* sp. LEA3 is grouped with *B. vietnamiensis* strain la1a4 isolated from rice in Peru. The taxonomy result provides relevance to the bacteria and type of habitat, which could be further linked to the biological function.

The ability of* Klebsiella* sp. LEA1 and *Pseudomonas* sp*.* LEA2 to grow on guaiacol, veratryl alcohol, and 2, 6-dimethoxyphenol (2,6-DMP) (Supplementary Table S2) suggests the presence of genes, pathways, or metabolisms that allow for oxidation of both phenolic and non-phenolic lignin monomers. The inability of *Burkholderia* sp. LEA3 to grow on guaiacol-supplemented media might be due to the toxicity of guaiacol^42,43^. Guaiacol is a predominant aromatic monomer found in depolymerized lignin^44^. It makes up parts of all lignin, especially softwood lignin^44,45^. Chow, et al.^41^ reported the incapability of *Streptomyces* sp. D7 to further degrade guaiacol after converting vanillic acid to guaiacol via decarboxylation. Enhancement of the oxidation of guaiacol by *Klebsiella* sp. LEA1 and *Pseudomonas* sp. LEA2 was observed when copper (100 μg/mL CuSO_4_) was supplied in the medium (Fig. 2A).

Copper has been shown to effectively enhance the production of ligninolytic enzymes. Furthermore, numerous bacterial multi-copper oxidases or laccases have been evaluated for their ability to act on both phenolic and non-phenolic compounds^38,46–50^. Therefore, it is plausible that *Klebsiella* sp. LEA1 and *Pseudomonas* sp. LEA2 may harbor copper-binding enzymes responsible for lignin utilization. Rezaei et al.^47^ reported a 13.7-fold increase in laccase production with the addition of 0.5 mM CuSO_4_ compared to the basal medium in *Aquisalibacillus elongatus*. Aside from its role in enzyme production, the supply of Cu^2+^ as an essential metal ion is pivotal for maintaining enzyme activity and overall lignin degradation efficiency. Copper serves as a cofactor in the active sites of laccases, facilitating the oxidation of lignin substrates^51^. Therefore, ensuring an adequate supply of copper ions in the growth medium is essential for maximizing the activity of ligninolytic enzymes and promoting efficient lignin degradation by bacteria.

The decline in aromatic absorbance at 280 nm indicated the reduction of the total aromatic components that occurred in the presence of *Klebsiella* sp. LEA1 and *Pseudomonas* sp. LEA2 during the 7-day incubation period. The higher growth rate and cell density of *Pseudomonas* sp. LEA2 may indicate its greater tolerance to lignin or better capability of lignin depolymerization and conversion. The decline in cell growth observed after day 3 of incubation is possibly due to the accumulation of phenolic compounds and other products that can produce inhibitory effects on cell growth^52^.

SEM images revealed rough surfaces and pores on the surface of lignin particles treated with isolates further indicating the potential lignin degrading ability of the isolates. Similar results have been reported in other studies that treated alkali lignin with lignin degrading bacteria^6,53^. During the process of the SEM sample preparation, the supernatant from control samples appeared colourless while the supernatant from bacteria treated samples was brownish. This phenomenon likely arises from the erosion of the lignin surface, leading to the detachment of smaller particles that are subsequently absorbed or extracted into ethanol. A similar observation was also noticed in the ethyl acetate extracts of control and bacteria treated samples for GC–MS analysis. Extracts of uninoculated samples were colourless while those of samples treated with bacteria were yellowish in colour (Supplementary Fig. 4). GPC analysis suggests that *Klebsiella* sp. LEA1 and *Pseudomonas* sp. LEA2 possess the capability to depolymerize lignin, resulting in the production of smaller lignin molecules. This is supported by the observed shift in the chromatogram peak, signifying a decrease in the Mw_,_ along with the accumulation of small lignin-derived molecules. It should be noted that the alkaline lignin used in this study underwent sterilization through autoclaving, a process wherein moist heat and pressure could potentially impact the structure of the alkaline lignin^54,55^. Therefore, we cannot rule out the possibility that the two isolates may also utilize the molecules degraded through abiotic degradation. Future studies aimed at assessing the utilization of small phenolic compounds, such as HPLC, could enhance precision in evaluating the degradation of lignin.

Many lignin-derived intermediates and products were detected by GC–MS analysis by day 7. It appears that most degradation products were formed or accumulated at the latter stage of incubation. Orellana, et al.^56^ reported that most up-regulation of ligninolytic enzymes such as laccase, peroxidases, and aryl-alcohol dehydrogenases occurred during the mid-exponential and early stationary growth phase in *Enterobacter lignolyticus* SCF1. Weinstein, et al.^57^ also reported the degradation of β-guaiacyl ether-linked lignin dimeric compounds during the stationary growth phase of *Phanerochaete chrysosporium*. Lignin-related compounds detected by GC–MS throughout 7 days of incubation under microaerobic conditions suggest that *Klebsiella* sp. LEA1 possibly used the catechol branch of the β-ketoadipate pathway for the metabolism of aromatic compounds obtained from alkali lignin (Fig. 5). Several lignin-related monomers such as cinnamic acid, catechol, benzoic acid and 4-hydroxybenzoic acid, which can be further degraded through the gentisate or β-ketoadipate pathways were detected after 7 days of incubation^58–60^. The presence of succinic acid (butanedioic acid) in the degrading samples suggests mineralization by *ortho-*cleavage of catechol through the β-ketoadipate pathway before finally entering the tricarboxylic acid (TCA) cycle for an energy source in *Klebsiella* sp. LEA1^61,62^. Our proposed mechanism of *Klebsiella* sp. LEA1 was in accordance with the genomic analysis of *Klebsiella pneumoniae* AWD5 which harbors an *ortho*-cleavage pathway for polyaromatic hydrocarbon degradation^63^. Although the metabolic pathway employed by *Pseudomonas* LEA2 cannot be established in this study, it is evident that the isolate is capable of utilizing lignin-based compounds, as inferred from the GC–MS and GPC analysis. Previous studies have demonstrated the coexistence of both protocatechuate and catechol branches within the β-ketoadipate pathway in *Pseudomonas aeruginosa* which are selectively used depending on the compounds or intermediates the bacterium is exposed to^64,65^.

Under aerobic incubation, most lignin-related compounds present in the samples were completely degraded by day 7 suggesting that oxygen could accelerate the degradation process in *Klebsiella* sp. LEA1. A previous study on *Phanerochaete chrysosporium* revealed enhancement of lignin dimer metabolism at increased oxygen concentrations suggesting the oxidative nature of steps involved in lignin degradation^25^. Vanillic acid has been identified as one of the major aromatic metabolites obtained from different intermediates and enzymatic reactions involved in lignin degradation. Vanillic acid can also be further degraded or mineralized to simpler compounds depending on the pathway employed by the bacteria^12,13,66,67^. As seen in Table 2 and Table S3, vanillic acid was present in samples throughout the incubation period. Although the degradation steps after the accumulation of vanillic acid are unclear based on the GC–MS result, it is possible that ligninolytic bacteria funnel vanillic acid into protocatechuic acid (Fig. 5). Protocatechuic acid is further dearomatized through the β-ketoadipate pathway by protocatechuate cleavage before finally entering the TCA cycle^12,60,67^.

Previous studies in *Klebsiella* and *Pseudomonas* have reported the presence of a complete β-ketoadipate pathway through the protocatechuate and catechol routes as well as genes and enzymes involved in lignin degradation such as peroxidases, monooxygenases, and laccase^63,68–71^. Discrepancies observed between alkali lignin degradation patterns under aerobic and limited oxygen conditions indicate the effect of environmental factors, particularly oxygen, on lignin metabolism. As seen in the results presented here, diverse degradation products were detected under microaerobic conditions compared to aerobic conditions, while vanillic acid and vanilonitrile were the only products detected under aerobic conditions. Many of the compounds discovered under oxygen limited conditions are valuable in the food, flavour and aroma industries as well as in both pharmaceutical and cosmetic sectors^12^. Thus, microaerobic conditions might be recommended to obtain a wider profile of compounds from bacterial lignin degradation. Although some studies have reported the ability of bacteria to degrade lignin, most of these studies mainly focus on lignin degradation under aerobic conditions^59,60^. Hence, bacteria that can utilize lignin under limited oxygen conditions and elevated temperature (40 °C) would be advantageous in anoxic conditions, which are more applicable in industrial settings. Further characterization of the physiological and biochemical features of these isolates, along with an understanding of related regulatory systems, may facilitate the optimization of lignin valorization for the production of various value-added compounds, particularly under oxygen-limited conditions.

## Materials and methods

### Soil sampling and bacterial isolation

Soil samples were collected at 20–30 cm depth from a deciduous forest in Nan province, Thailand^32^. Sample collecting sites in this study located in the boundary area of the national park, no restrictions regarding permission are required. Samples were immediately kept in microaerobic conditions (CO_2_:5–6%; O_2_:6–10%) using AnaeroPack™ (Mitsubishi Gas Chemical, Japan) and stored at 4 °C for further analysis within two days. To isolate lignin degrading bacteria, the soil samples were enriched in autoclaved minimal salt media (MSM) containing 1 g/L alkali (Sigma-Aldrich) lignin (L-MSM) as the sole carbon source at 40 °C for 7 days under microaerobic conditions. The L-MSM contained (g/L) Na_2_HPO_4_ (2.4), K_2_HPO_4_ (2.0), NH_4_NO_3_ (0.1), MgSO_4_.7H_2_O (0.5), CaCl_2_ (0.01), MnSO_4_.H_2_O (0.02), FeSO_4_.7H_2_O (0.01), alkali lignin (1.0), pH 7.2. The L-MSM was sterilized by autoclaving before use. After the enrichment period, 1 mL of culture was serially diluted and spread on Berg’s mineral salts agar supplemented with 1% carboxymethyl cellulose (CMC) (g/L) (NaNO_3_ (2), K_2_HPO_4_ (0.05), MgSO_4_.7H_2_O (0.5), MnSO_4_.H_2_O (0.02), FeSO_4_.7H_2_O (0.01), CaCl_2_.2H_2_O (0.02). Isolates that appeared on CMC plates were restreaked four times to get axenic cultures and finally transferred to plating on L-MSM agar.

### 16S rDNA identification of isolates

Bacterial isolates that grew on L-MSM were cultured overnight in LB medium at 40 °C, with periodic shaking at 200 rpm. Genomic DNA was extracted from overnight cultures using the phenol–chloroform extraction method. The full length of 16S rDNA was amplified using universal primers 27F and 1492R (27F 5’-AGAGTTTGATCCTGGCTCAG-3’; 1492R 5’-GGTTACCTTGTTACGACTT-3’) followed by sequencing analyses. For bacterial identification, sequence homology was compared using the Basic Local Alignment Search Tool (BLAST) against the National Center for Biological Information (NCBI) database. A phylogenetic tree of 16S rDNA was constructed by the neighbor joining method, 1000 bootstrap replications using MEGA11 software.

### Growth on different lignin monomers

The ligninolytic ability of selected isolates was tested using model lignin monomers (guaiacol, veratryl alcohol, and 2, 6-dimethoxyphenol (2,6-DMP)) (Sigma-Aldrich). Guaiacol and 2, 6-DMP represented phenolic substrates, while veratryl alcohol represented a non-phenolic substrate^61^. Preculture of bacterial isolate in LB with the OD_600_ of 1.0 was collected after centrifuging at 5000 × *g* for 5 min and washed with normal saline (0.9% NaCl) three times. The bacterial suspension was then adjusted to OD_600_ of 0.1 and 5 µl of the suspension was gently dropped onto MSM plates supplemented with 0.1% (v/v) guaiacol, veratryl alcohol, and 0.1% (w/v) 2, 6-DMP. The MSM plates were incubated for 7 days at 40 °C under microaerobic conditions.

### Effect of copper (Cu) on lignin degradation

0.1 g/L CuSO_4_ was incorporated into LB agar and MSM agar supplemented with 0.1% (v/v) guaiacol to investigate the influence of copper on guaiacol oxidation. The sample inoculated with *Klebsiella* sp. LEA1 and *Pseudomonas* sp. LEA2 were kept at 40 °C in microaerobic conditions for 7 days. Agar plates were examined for the oxidation of guaiacol indicated by the presence of pink or brown pigments around bacterial colonies.

### Growth characteristic of the isolates and total phenolic content analysis

Based on the effect of copper on guaiacol oxidation, L-MSM was modified to contain 0.1 g/L CuSO_4_. *Klebsiella* sp. LEA1 *Pseudomonas* sp. LEA2 and were inoculated into 50 mL L-MSM at an equivalent OD_600_ of 0.1. Growth of the bacterial isolates in the media over time was followed at 40 °C for 7 days under microaerobic conditions. The culture suspension was sampled on days 1, 2, 3, 5, and 7 of the incubation to determine colony forming units (CFU), as the colour of alkali lignin interfered with the turbidity of the solution.

To further test for the reduction of total phenolic content, the culture supernatant was withdrawn and acidified with concentrated hydrochloric acid (HCl) to pH 2. The sample was centrifuged at 12,000 × *g* for 10 min and the supernatant was collected. Total phenolic content was monitored by measuring the remaining phenolic compounds at the absorbance of 280 nm using a V530 UV/Vis spectrophotometer (Jasco, Japan).

### Field emission scanning electron microscope (FE-SEM)

Visual assessment of the surface morphology and structure of lignin samples was done using a field emission scanning electron microscope (FE-SEM). Two milliliters of control (no bacterium) and inoculated samples were withdrawn after 7 days and centrifuged at 12,000 × *g* for 10 min. The precipitates were collected and fixed in 2.5% glutaraldehyde. Precipitates were washed twice in phosphate buffer saline (PBS) pH 7 followed by sterile distilled water. The samples were dehydrated in a gradient of 25–95% ethanol and coated with a layer of Pt/Pd alloy via critical point drying and ion sputtering. Afterward, samples were imaged with FE-SEM (Hitachi SU-8010).

### Gel permeation chromatography (GPC)

The molecular weight distribution of alkaline lignin was investigated using the GPC-Tetrahydrofuran (THF) system (Waters e2695, Netherlands). A total of 0.1% of alkaline lignin was incubated in a minimal salt medium with or without bacteria at 40 °C for 7 days. Alkaline lignin in the media was collected by adding 6 M HCl, centrifuged at 12,000 × *g* for 10 min, and dried at 60 °C. Dried lignin was acetylated using 2 mL of equal volume of acetic anhydride and pyridine. The reaction mixture was stirred for 24 h before adding 0.1 M HCl, centrifuged at 12,000 × *g* for 10 min, and dried at 60 °C. Dried acetylated samples were dissolved in THF and filtered through 0.2-μm nylon membrane syringe filters. Size exclusion was performed at room temperature with THF as the mobile phase (flow rate of 1 mL min^–1^) with RI, light scattering and viscometry detectors. The calibration was performed by using polystyrene standards with the Mw range between 1250 and 920,000 g mol^−1^. For GPC analysis, three independent experiments were conducted.

### Gas chromatography mass spectrometry (GC–MS) analysis of lignin degradation products

*Klebsiella* sp. LEA1 and *Pseudomonas* sp. LEA2 isolates were grown in L-MSM under microaerobic conditions for 7 days in triplicates, while the investigation under aerobic conditions was additionally done in *Klebsiella* sp. LEA1. Samples were collected on days 3 and 7 of incubation. Fifty millilitres of samples were centrifuged (5000 × *g*, 30 min) and the supernatants were acidified to pH 1–2 using 6 M HCl. After acidification, samples were centrifuged and the supernatants were harvested. Supernatants were thoroughly extracted three times with ethyl acetate and the organic layer was collected followed by dewatering over anhydrous Na_2_SO_4_ in filter paper to remove moisture. The organic layer of triplicates of each treatment was pooled together, and samples were placed in a rotary evaporator at 37 °C for further moisture removal. The residues were then resuspended in 10 mL of ethyl acetate. Two milliliters of ethyl acetate suspension were derivatized after evaporation of the solvent under nitrogen gas using 70 µL N-Methyl-N-(trimethylsilyl) trifluoroacetamide with 1% trimethylchlorosilane (MSTFA) and 40 µL Methoxamine (MOX) reagent. The mixture was heated at 40 °C for 1 h with periodic shaking.

One microliter of the sample was subjected to the GC–MS (Agilent Technologies USA 5977B MSD; 7890 BGC) for separation by HP-5MS UI capillary column (29.90 m × 0.25 mm × 0.25 µm). Helium was used as a carrier gas with a flow rate of 1 mL/min. The column was held at 50 °C for 5 min and increased to 300 °C (10 °C per min, hold time: 5 min). The transfer line and ion source temperatures were maintained at 200 and 250 °C. A solvent delay of 3.5 min was selected. Electron ionization mass spectra in the range of 30–550 (*m/z*) were recorded at an electron energy of 70 eV. The compounds were identified by comparing the retention times of standards and data in the National Institute of Standards and Technology (NIST) library.

## Conclusion

In this study, bacteria capable of converting and utilizing alkaline lignin were isolated from soil samples collected from the forest in northern Thailand. *Klebsiella* sp. LEA1 and *Pseudomonas* sp. LEA2 were able to grow on alkali lignin and lignin monomers under microaerobic conditions and elevated temperature (40 °C). SEM images revealed the erosion of the lignin surface by the isolates, consistent with the depolymerization ability suggested by GPC analysis. The profile of compounds detected using GC–MS indicated the mineralization of lignin monomers and the possible presence of lignin-degrading enzymes, genes, and pathways such as the gentisate pathway and the β-ketoadipate pathway in *Klebsiella* sp. LEA1. Our results also indicate increased alkaline lignin conversion during the late stage of the incubation period of bacterial isolates. *Klebsiella* sp. LEA1 and *Pseudomonas* sp. LEA2 possess the ability to utilize alkaline lignin under the limited oxygen conditions commonly encountered in fermentation systems. Thus, the use of these lignin-utilizing bacterium can be explored further in studies related to lignin valorization and degradation under oxygen limited conditions.

### Supplementary Information

Supplementary Information.

## Data Availability

Raw data generated and analyzed in this study are available in the depository links: Bacterial growth: 10.6084/m9.figshare.21814164 GC–MS: 10.6084/m9.figshare.21814152 SEM images: 10.6084/m9.figshare.21958379 GPC: 10.6084/m9.figshare.25901380.v1 Requests for further information or materials should be addressed to P.H.
