# Supplementary material for: Evaluating lignin degradation under limited oxygen conditions by bacterial isolates from forest soil
Source: Sci Rep. 2024 Jun 10;14:13350. doi: 10.1038/s41598-024-64237-8 (PMC11164938; doi:10.1038/s41598-024-64237-8)
Supplement: Supplementary file 1 — Supplementary Information. [file 41598_2024_64237_MOESM1_ESM.pdf]

Evaluating lignin degradation under limited oxygen conditions by bacterial isolates from forest soil

Thitinun Sumranwanich[1], Esther Amosu[1], Surang Chankhamhaengdecha[1], Tanaporn Phetruen[2], Wethaka Loktumraks[1], Puey Ounjai[1], Phurt Harnvoravongchai[1]*

[1] Department of Biology, Faculty of Science, Mahidol University, Thung Phaya Thai, Ratchathewi, Bangkok 10400, Thailand
[2] Department of Biochemistry, Faculty of Science, Mahidol University, Thung Phaya Thai, Ratchathewi, Bangkok 10400, Thailand

Supplementary information

Table S1. Identification of bacterial isolates collected from forest soil

Isolate	Top hit strain	Accession no.	Identity
LEA1	*Klebsiella variicola* strain F2R9	NR_025635.1	99.93%
N1	*Klebsiella variicola* strain F2R9	NR_025635.1	99.85%
N2	*Klebsiella variicola* strain F2R9	NR_025635.1	99.78%
N3	*Klebsiella variicola* strain F2R9	NR_025635.1	99.71%
T4	*Klebsiella variicola* strain F2R9	NR_025635.1	99.49%
T5	*Klebsiella variicola* strain F2R9	NR_025635.1	99.93%
T6	*Klebsiella variicola* strain F2R9	NR_025635.1	99.70%
T7	*Klebsiella variicola* strain F2R9	NR_025635.1	99.78%
T8	*Klebsiella variicola* strain F2R9	NR_025635.1	99.78%
LEA2	*Pseudomonas aeruginosa* strain DSM 50071	NR_117678.1	100%
A1	*Pseudomonas aeruginosa* strain DSM 50071	NR_117678.1	100%
A2	*Pseudomonas aeruginosa* strain DSM 50071	NR_117678.1	100%
LEA3	*Burkholderia vietnamiensis* strain TVV75	NR_118872.1	99.93%
B1	*Burkholderia vietnamiensis* strain TVV75	NR_118872.1	99.93%
B2	*Burkholderia vietnamiensis* strain TVV75	NR_118872.1	99.93%
B3	*Burkholderia vietnamiensis* strain TVV75	NR_118872.1	100%

Table S2. Bacteria growth on MSM agar supplemented with different lignin monomers. The isolates were inoculated on MSM containing different lignin monomers: 0.1% v/v guaiacol, 0.1% v/v veratryl alcohol, and 0.1% w/v 2, 6-DMP for 7 days at 40ºC under microaerobic conditions.

**Bacteria**	**Growth ability**		
	**Guaiacol**	**Veratryl alcohol**	**2,6 DMP**
***Klebsiella* sp. LEA1**	+	+	+
***Pseudomonas* sp. LEA2**	+	+	+
***Burkhloderia* sp. LEA3**	ND	+	+

+: growth on medium; ND: no detection of growth. Experiment on bacterial growth was performed in triplicate.

Table S3: GC-MS product analysis of control and degraded samples under aerobic conditions. *Klebsiella* sp. LEA1 was grown in L-MSM under aerobic conditions at 40ºC for 7 days. **"+"**, compound was detected; **"-"**, compound was not detected in culture; RT: Retention time.

**No.**	**RT**	**Compound**	**Control**		***Klebsiella. sp.* LEA1**	
			**Day 0 and 3**	**Day 7**	**Day 3**	**Day 7**
1	11.257	Guaiacol	+	+	+	-
2	13.908	Benzeneacetic acid	+	+	-	-
3	14.335	Guaiacol, 4-ethyl-	+	+	-	-
4	14.845	4-Vinyl guaiacol	+	+	-	-
5	16.030	Vanillin	+	+	-	-
6	16.537	Vanilonitrile	-	-	+	+
7	17.727	Homovanillyl alcohol	+	+	-	-
8	18.113	Vanillic acid	+	+	+	+
9	19.119	Dihydro - coniferyl alcohol	+	+	+	-
10	27.206	Phenol, 2,2'-methylenebis[6-(1,1-dimethylethyl)-4-ethyl	+	+	+	+

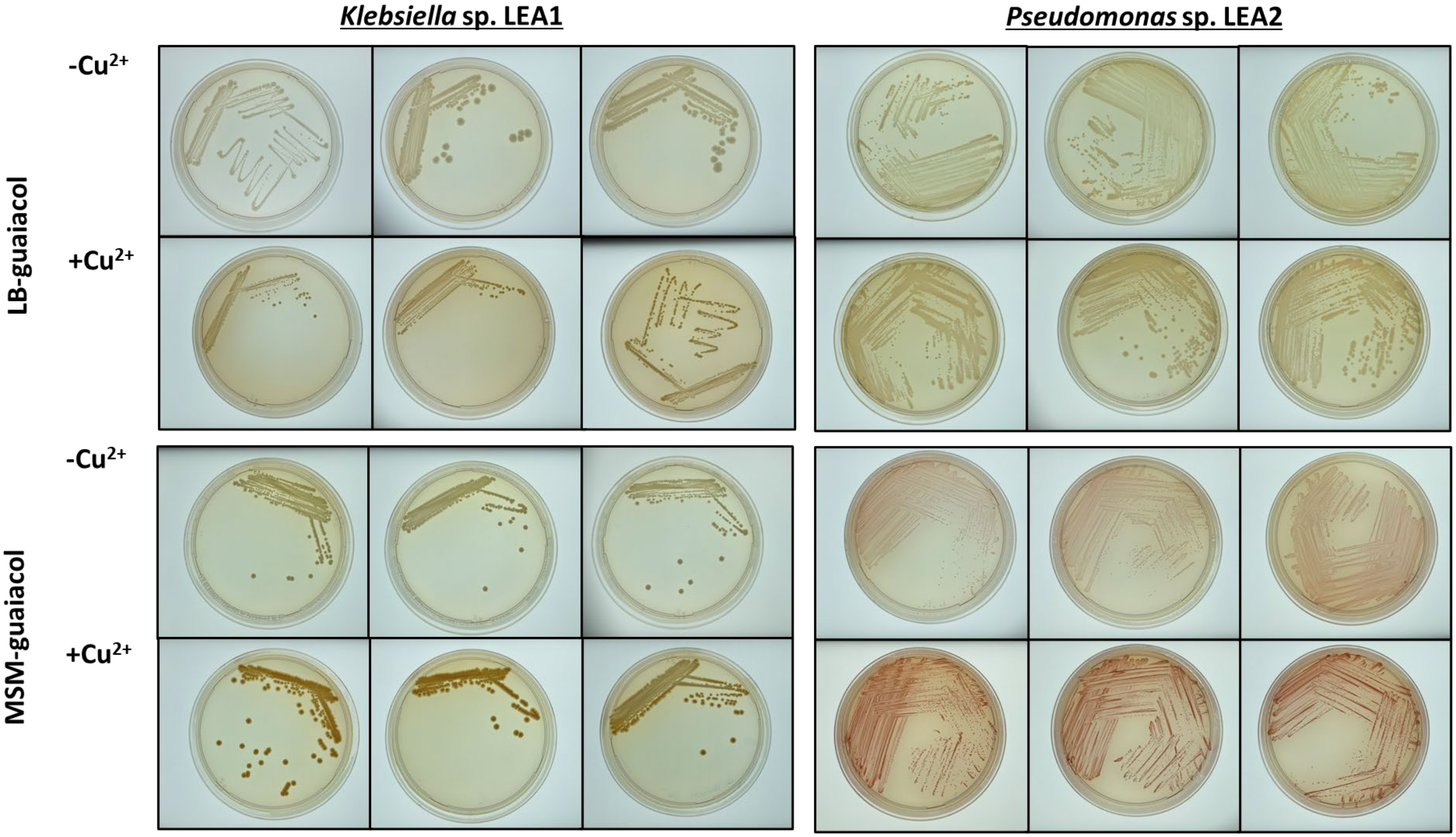

Supplementary Figure 1: Guaiacol oxidation on minimal salt media (MSM) and LB media supplemented with 0.1% (v/v) guaiacol represented in triplicated

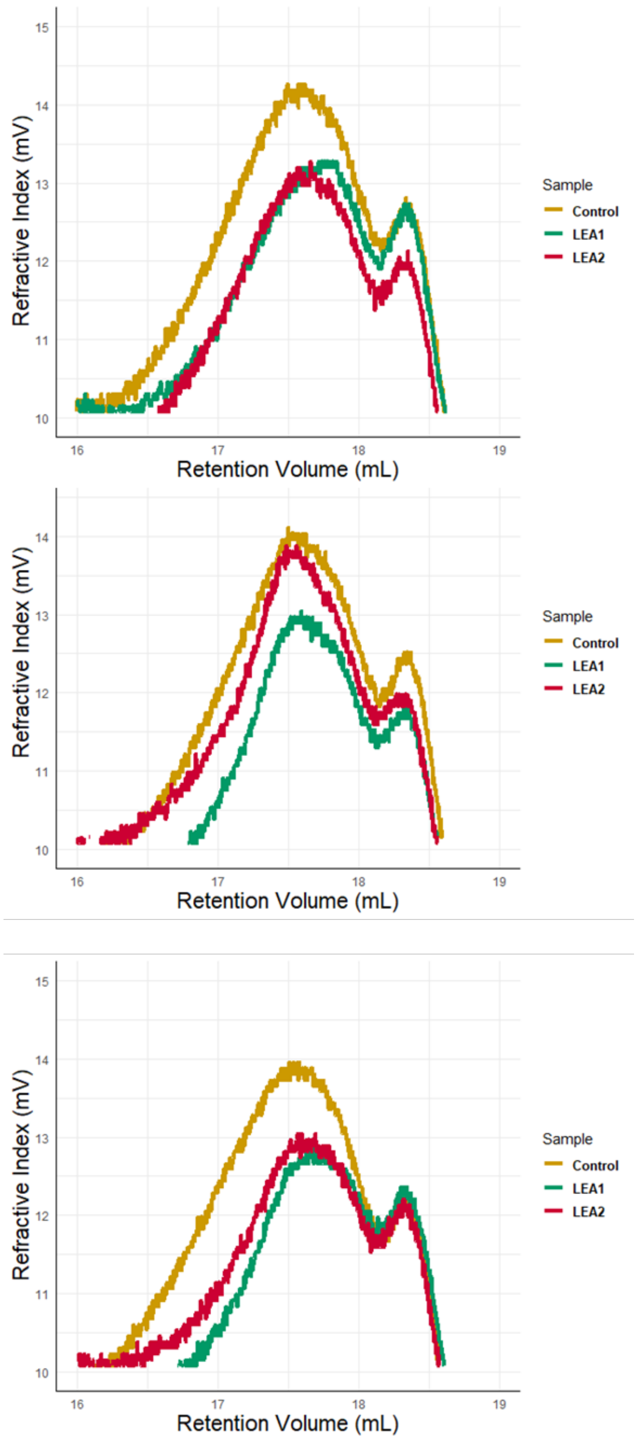

Supplementary Figure 2: Individual GPC chromatogram constituted from the data of each replication

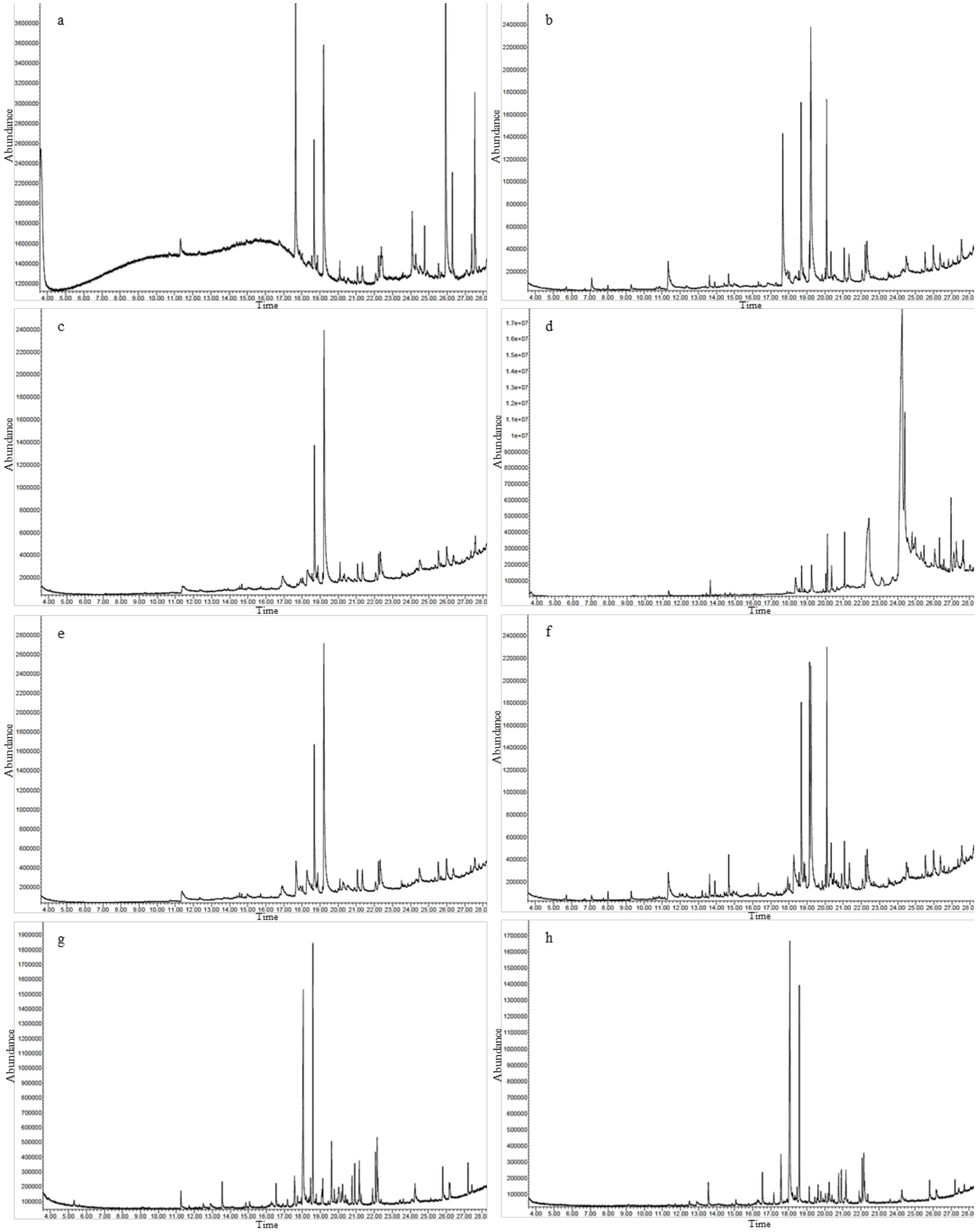

Supplementary Figure 3: GC-MS chromatogram profile of ethyl acetate extracts from control samples day 0(a); day 7 (b); and samples inoculated with bacteria under microaerobic condition *Pseudomonas* sp. LEA2 day 3 (c); day 7 (d); *Klebsiella* sp. LEA1 day 3 (e); day 7 (f); samples inoculated with *Klebsiella* sp. LEA1 under aerobic conditions day 3 (g); day 7 (h)

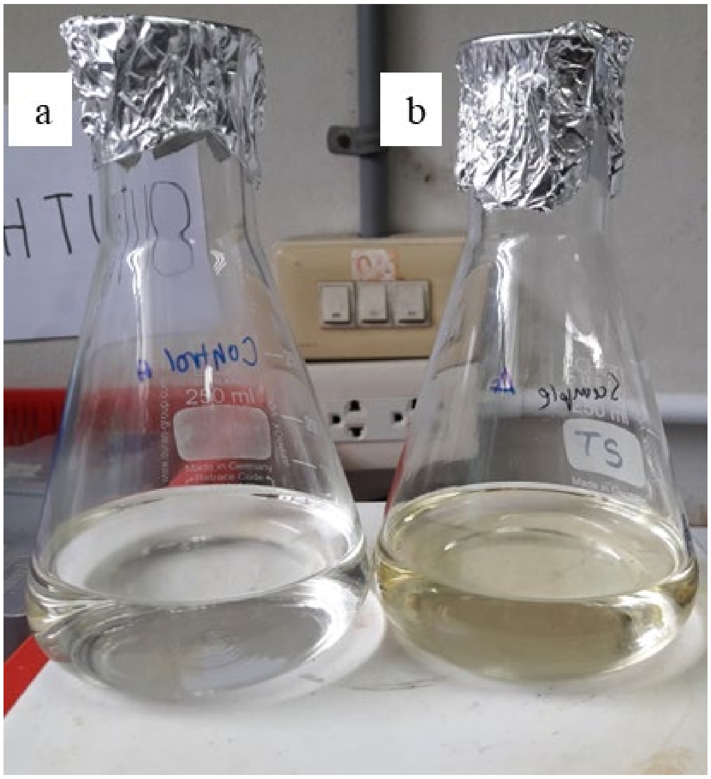

Supplementary Figure 4: Ethyl acetate extracts from (a) Uninoculated lignin control samples; (b) Inoculated lignin sample

Data availability

Raw data of growth and degradation kinetic: https://dx.doi.org/10.6084/m9.figshare.21814164

Raw data of GC-MS: https://dx.doi.org/10.6084/m9.figshare.21814152

SEM images: https://dx.doi.org/10.6084/m9.figshare.21958379
